# 2025 Acknowledgment of *Microbiology Spectrum* Reviewing Editors

**DOI:** 10.1128/spectrum.00756-25

**Published:** 2025-05-06

**Authors:** Christina A. Cuomo

**Affiliations:** 1 Brown University 6752, Providence, Rhode Island, USA

## EDITORIAL

The Reviewing Editor program at *Microbiology Spectrum* is open to early-career microbiologists from across the globe. The Reviewing Editor role is distinct from the Editor role in that while Editors choose peer reviewers and make decisions on manuscripts, the Reviewing Editors serve as expert reviewers for one to two papers per month (a maximum of 24 per year) and receive feedback on their reviews. Reviewing Editors advance to the Editor role based on their performance and subject area expertise needs for Editors at the journal.

Currently, we have over 120 Reviewing Editors from 31 countries with expertise across microbiology. In the past year, our Reviewing Editors provided over 500 reviews for the journal—extraordinary service for which we are very grateful. Furthermore, 33% of all manuscripts were evaluated by at least one Reviewing Editor.

This past year, we continued to promote Reviewing Editors to the Editor role. These individuals were selected based on their subject area expertise, number of reviews completed, and the quality of the reviews.

We thank all our Reviewing Editors, past and present, for being a part of this program. Please join us in congratulating the following people who served in the Reviewing Editor role in 2024 (Reviewing Editors who were promoted to the Editor role are bolded):

Rachy Abraham

Nurul Ahmad

**Philips Akinwole**

**Cassio Almeida-da-Silva**

Megan Amerson-Brown

Jorge Arca-Suárez

**Charina Gracia Banaay**

Arden Baylink

Michael Becker

Francesca Benedetti

Banhi Biswas

Gillian Bruni

**Irene Burckhardt**

Weiwei Cai

Yi Cai

Amanda Calvert

Victoria Campodonico

Cecilia Carvalhaes

Alexandra Chiaverini

Vito Colella

Francesco Comandatore

Jôice Corrêa

Sneha Couvillion

Maria De Francesco

Kanan Desai

Bismarck Dinko

**Alex Dulovic**

Erin Eggleston

Jia-Yih Feng

Madiha Fida

Carlos Figueroa Castro

Hui-Chen Foreman

**Estela Galvan**

Ketaki Ganti

Miguel Angel Garcia Bereguiain

Emilia Ghelardi

Payal Gupta

Mehrad Hamidian

**Shannon Hinsa-Leasure**

Laura Huber

Yosef Huberman

Kelly Ishida

**Juan Jaworski**

Hyung Jong Jin

Teny John

Lovleen Joshi

Denis E. Kainov

Jaspreet Kaur

Johanna Kenyon

Otmane Lamrabet

Dexi Li

Wentao Li

**Yongxin Li**

Xiaoyan Lin

Amelia Lindsey

**Hao-Yu Liu**

Qin Liu

Veranja Liyanapathirana

Ti Lu

Sonia Luque

Mor Lurie-Weinberger

Tsidiso MAPHANGA

Jose Martinez-Navio

Bruno Martorelli Di Genova

**Patrick McDaneld**

Jun Miao

Sheema Mir

Sarita Mohapatra

Walaa Mousa

Shahzad Munir

Azdayanti Muslim

Jowita Samanta Niczyporuk

**Michael Owusu**

Chin-Soon Phan

Cécile Philippe

Parmanand Prabhakar

**Andrea Prinzi**

Elias Rahal

Ashok Reddy

Christopher Rice

Emanuela Ruggiero

Sanghamitra Saha

Jennifer Salerno

Garima Singh

**Nicolas Soler**

Jaime Soria

Katie Summers

Hao Tan

Fang Tang

Li Tao

Anuraj Theradiyil Sukumaran

Sangeeta Tiwari

Maria Tomas

Xinzhao Tong

Deeksha Tripathi

Gareth Trubl

Pablo Tsukayama

Filipa Vale

**Vanessa Varaljay**

Ravindra Veeranna

Musturi Venkataramana

Benjamin Von Bredow

Crista Wadsworth

Caray Walker

Fang Wang

Wei Wang

Yan Wang

Yi Wang

**Michael Whitfield**

**Natalie Whitfield**

Zhu Xiaoyu

Yifei Xu

Pragya Yadav

**Srujana Samhita Yadavalli**

Feng Yang

**Rebecca Yee**

Jinki Yeom

Yongchao Yin

**Kayvan Zainabadi**

Krzysztof Zawierucha

Changyi Zhang

Jianhua Zhang

Xue Zhang

Zhemin Zhang

Zhen Zhang

Yuchao Zhao

Chunyi Zhou

